# *Schizosaccharomyces pombe* as a predictor toxicity tool

**DOI:** 10.1016/j.mex.2024.102823

**Published:** 2024-06-24

**Authors:** Consuelo Álvarez-Herrera, Sara Maisanaba, María Llana Ruíz-Cabello, Guillermo Repetto

**Affiliations:** Area of Toxicology, Universidad Pablo de Olavide, Sevilla 41013, Spain

**Keywords:** Yeast, Toxicity, Dose-response, *Schizosaccharomyces pombe* as a predictor toxicity tool

## Abstract

The fission yeast *Schizosaccharomyces pombe* is frequently used as a genetically manipulable model system, offering valuable understandings into cellular mechanisms. In the present study, a comprehensive step-by-step methodology for the research of the action mechanisms and detoxification by efflux pumps is showed. The protocol involves the thawing and culture of yeast cells in liquid medium under controlled conditions to ensure exponential growth. After that, a dose-response assessment is carried out by culturing wild-type cells in liquid medium, followed by exposure to increasing concentrations of the toxic substances. Optical density measurements are taken spectrophotometrically after exposure, and the process is repeated at least three times for quantitative analysis. Subsequently, defective mutants are selected to explore specific mechanisms of action or detoxification by efflux pumps, with cultures prepared and treated similarly to the wild type. Optical density measurements are again taken after exposure for quantitative analysis. This methodology ensures robust and reproducible results for the research toxic substances effects on *S. pombe*.

- *Schizosaccharomyces pombe* is an adequate tool to evaluate contaminants toxicity.
- Dose-responses curves are obtained on wild type to evaluate toxicity mechanisms.
- This methodology ensures robust and reproducible results for the research toxic substances effects on *S. pombe.*

## Graphical abstract

**Figure:**
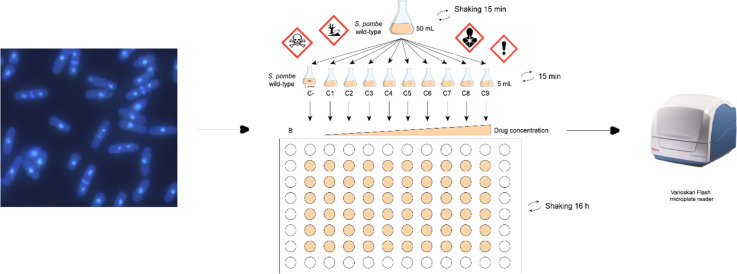

Specifications table

**Table:** 

Subject area:	Pharmacology, Toxicology and Pharmaceutical Science
More specific subject area:	Toxicology
Name of your method:	Schizosaccharomyces pombe as a predictor toxicity tool
Name and reference of original method:	N.A
Resource availability:	N.A

## Background

The fission yeast *S. pombe* is a eukaryotic ideal model organism for discovering chemical inhibitors and analyzing drug mechanisms of action by studying its defective strains [8]. For all of that, it has been decided to search for a method that provide us relevant information in a few hours about cytotoxicity, action mechanisms and pumps detoxication generated by several daily drugs. Here, it has been proposed a procedure based on two differentiated steps. In the first one, a dose-response assessment is completed in the wild type strain and in the second one, the investigation of the main mechanisms of action or detoxification by efflux pumps is carried out in the defective strains (Fig. 3).

## Method details

### Key resources TABLE

The S. pombe strains were provided from the Centro Andaluz de Biología del Desarrollo (CABD), from R.R. Daga´s and S. Moreno´s labs (Tables 1-Table 2, Table 3).

**Table 1:** Strains of *Schizosaccharomyces pombe* as a predictor toxicity tool.

Strain	Genotype	Code
*Wild-type*	h-	RD1317
*mph1Δ* (Mps1p-like homolog)	h- *mph1::ura4+ leu1.32*	RD981
*pmk1Δ* (human ERK1/2 ortholog)	h- *pmk1::ura4+ leu1.32 ura4-D18* -	RD1000
*sty1Δ (mammalian p38 ortholog)*	h- *sty1::ura4+ ura4-D18*	RD1105
*pmk1Δsty1Δ*	*h*+ *ade- leu 1.32 ura4-D18 sty1::ura4+ pmk1-HA6H::ura4+*	RD1525
*rad3Δ* (ataxia telangiectasia and Rad3-related human ortholog)	*h*+ *rad3::ura4+ ade6-M210 leu1.32 ura4-D18*	RD2247
MDR-sup (*pap1Δ, bfr1Δ, pmd1Δ, mfs1Δ and caf5Δ)*	*h*+ *pap1::kanR bfr1::hygR pmd1::natR caf5::kanR mfs1::natR ade6-M216 leu1.32*	RD3950
*pap1Δ* (c- Jun homolog)	h- *pap1::ura4+ leu1.32 ura4-D18*	RD1725
*bfr1Δ* (human P-glycoprotein homolog)	*h*+ *bfr1::kanR ade6- M210 ura4-D18 leu1.32*	RD2137
*pmd1Δ* (human P-glycoprotein homolog) (regulated by pap1 and sty1)	*h*+ *pmd1::kanR ade6-M210 ura4-D18 leu1.32*	RD2140
*mfs1Δ*	*h*+ *mfs1::ura4+ ade6-M210 ura4-D18 leu1–32*	RD2304
*caf5Δ*	*h+ caf5:ura4+ ade6-M210 ura4-D18 leu1–32*	RD2305

**Table 2:** Reagents for yeast growth media (YES).

Reagent	Concentration	Source	Identifier
Yeast extract	5 g/L in ddH_2_O	Fisher scientific	1196–5365
Glucose	30 g/L in ddH_2_O	Fisher chemical	G/0500/53
L-Leucine	0.3 g/L in ddH_2_O	Sigma Aldrich	101,493,398
Uracil	0.3 g/L in ddH_2_O	Amresco	0975
L-Histidine	0.3 g/L in ddH_2_O	Acros organics	411,730,250
Adenine	0.3 g/L in ddH_2_O	Amresco	0183
Agar*	2 % (w/v) in ddH_2_O	Fisher scientific	1205–3756

⁎ Only for agar plates.

**Table 3:** List of equipment and material.

Equipment	Source	Identifier
96-well multidish	Thermo Fisher Scientific	H2861301885
Autoclave	Selecta Presoclave II 80	4,001,759
Vertical laminar flow	Thermo Scientific MSC advantage	51,025,411
Incubator shaker	Thermo Scientific MaxQ 8000	SHKE8000
Microplate reader	Varioskan Flash, Thermo Fisher Scientific	VLBL00GD2
Petri dishes 90 mm diameter	Gosselin	SB90–101
Plate shaker	Minishaker PSU-2T bioSan	01,015,514,100,506
Spectrophotometer	Thermo Scientific Multiskan go microplate spectrophotometer	51,119,200
Sterile flasks 25 mL	Labbox	EFNG-025–010
Sterile flasks 100 mL	Fisherbrand	FB33139
Sterile flasks 250 mL	Fisherbrand	FB33140
Stove	Memmert Thermo Fisher Scientific	12,686,977

### Step-by-step method details

A. **Preparing the experimental model**

*1. S. pombe* yeast cells are thawed on agar plates and incubated in a stove for 48 h at 25 °C to initiate their growth.
2. A pre-inoculum is prepared by taking a small amount of biomass from the plate and putting it into 5 mL of YES medium in a sterile flask with a capacity of 25 mL.^1^
3. The flask with the yeast pre-inoculum is cultured for at least two generation times (2–3 h) with continuous shaking at 180 rpm in an incubator shaker at 25 °C.
4. The optical density is measured at 595 nm (or the cells counted) by adding 100 µL in a well of a 96-well microplate to assure the correct growth (about 0.08–0.1). The culture is diluted in YES about 1:4 if the time to growth is 8 h and about 1:10 if the time is 16 h.
5. This process must be repeated at least two times before starting the assay.^2^

B. **Dose-response assessment**

6. The optical density is measured, and the culture is diluted to 0.004 OD with a final volume of 55 mL in a 250 mL flask.
7. The flask is shaken in the incubator shaker at 180 rpm and 25 °C for 15 min.
8. The content of the flask is divided into 10 flasks, each containing 5 mL of YES.
9. To the first flask, drug is not added as it will serve as a negative untreated control group.
10. Increasing concentrations of the toxic substance under study are added to flasks 2 through 10, maintaining the same dilution /solvent concentration.^3^".
11. The flasks are shaken in the incubator shaker at 180 rpm and 25 °C for 15 min.
12. The content of the flasks is transferred to a 96-well plate. Each well B-F is filled with 200 µL of the corresponding exposure solutions (6 wells per concentration). Thus, column 1 is filled with YES medium and it serves as the blank control, column 2 is filled with the content of flask 1, column 3 with flask 2, and so on until column 10. The remaining wells along the edges should be filled with YES or distilled water to maintain humidity.
13. Place the 96-well plate under constant agitation at 1200 rpm on a plate shaker for 16 h at 25 °C in a humidity chamber.
14. After the period of exposure, the density of the cell populations is measured spectrophotometrically using a microplate reader Varioskan Flash^4^ (Fig. 1).

**Fig. 1:**
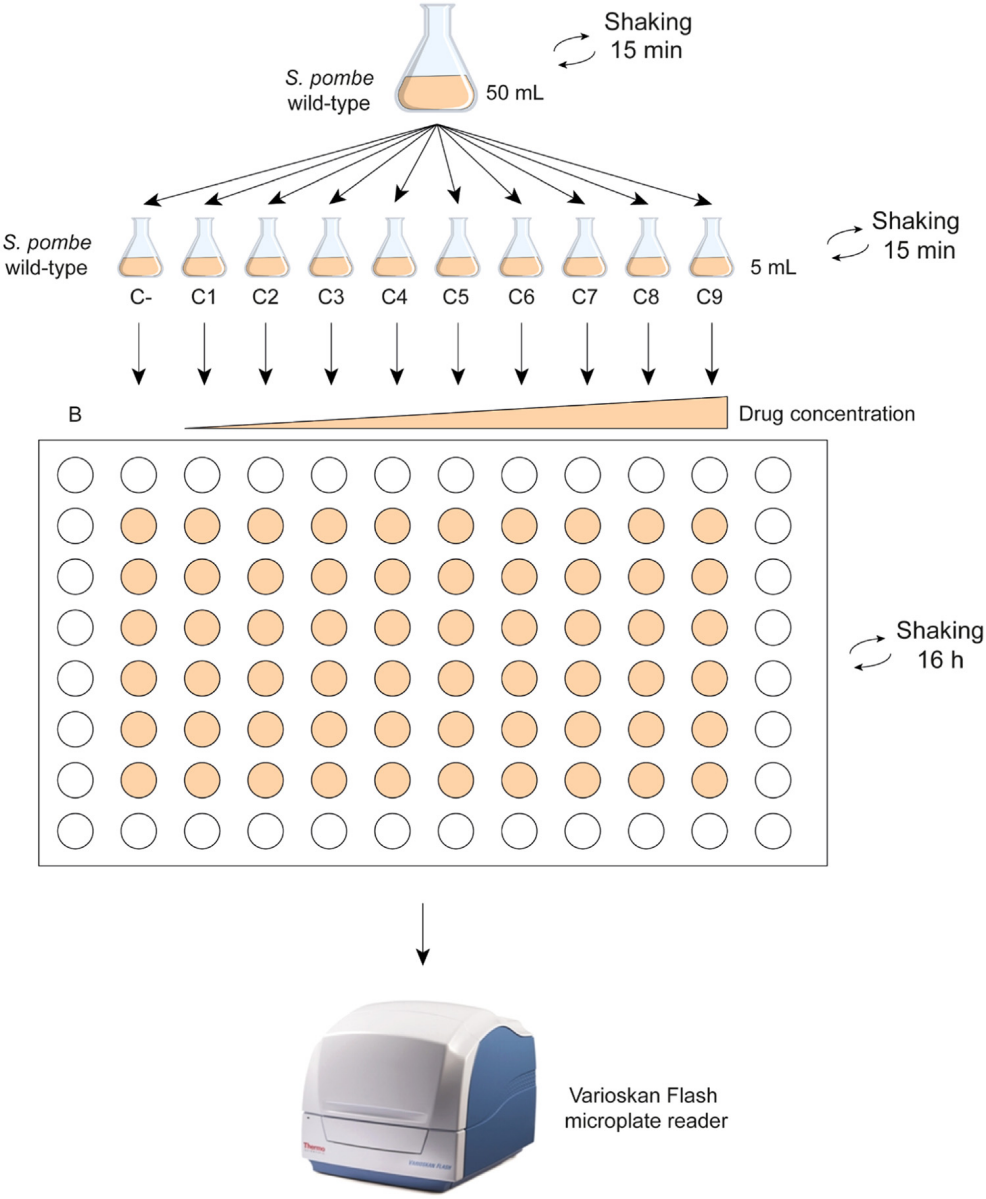
Dose response assessment. Schematic illustrations of the steps 7–15. Experimental groups: B, blank (only YES medium); C-, negative untreated control; C1 to C9: exposure solutions in increasing concentrations of the assayed compound.
15. Each experiment is repeated at least three times.
16. Quantitative analysis of the values is performed, considering that OD values are correlated to the number of *S. pombe* cells. Experimental groups are statistically compared to the untreated cells.
17. A dose-response regression is represented with Excel or other software [1,2].

C. **Investigation of the main mechanisms of action and detoxication by efflux pumps**

18. After the dose response analysis, two concentrations that showed from 20 to 50 % growth inhibition in the wild-type strain are selected (EC80, EC50) to research the main mechanisms of action of the chemical or the detoxication by efflux pumps. Depending on the type of study, specific defective strains should be select (Table 4, Table 5).

**Table 4:** Strains used for the study the mechanisms of action.

Strain	Hypersensitive to
*Wild-type*	Basal control
*mph1Δ*	Extremely marked specificity for microtubule or spindle defects [3]
*pmk1Δ*	Global sensitivity to chemicals causing cellular stress [4,5]
*sty1Δ*	Extremely marked specificity and sensitivity for oxidative stress. High sensitivity to osmotic stress [6]
*pmk1Δsty1Δ*	Extremely marked specificity for oxidative stress and very high sensitivity to osmotic stress. Similar sensitivity as *sty1Δ*, but with some components due to *pmk1* deletion [5]
*rad3Δ*	Extremely marked specificity for DNA damage. Very high sensitivity for oxidative stress [7]

**Table 5:** Strains used for the study of detoxication by efflux pumps.

Strain	Hypersensitive to
*Wild-type*	Basal control
MDR-sup (*pap1Δ, bfr1Δ, pmd1Δ, mfs1Δ and caf5Δ)*	Substances excluded by Caf5, Pmd1, Bfr1 and Mfs1 pumps. Very high sensitivity to microtubule interactions, oxidative stress, and DNA damage. Proliferation stimulated by low-intensity hyperosmotic stress [8]
*pap1Δ*	Extremely marked specificity for oxidative stress, but with less sensitivity than *sty1Δ* cells [9]
*bfr1Δ*	Sensitivity to substances excluded by the Bfr1 transporter [10]
*pmd1Δ*	Sensitivity to substances excluded by the Pmd1 transporter [11]
*mfs1Δ*	Sensitivity to substances excluded by the Mfs1 transporter. Possible stimulation of proliferation by DNA damage [12]
*caf5Δ*	Sensitivity to substances excluded by the Caf5 transporter. High sensitivity to oxidative and hyperosmotic stress. Possible stimulation of proliferation by DNA damage [12]
19. Once the defective mutants are selected, yeast are cultured at least two times to exponential growth after the pre-inoculum. The process is also carried out with the wild type.
20. Subsequently, the OD is determined, and the cultures are diluted to 0.004 OD, reaching a final volume of 18 mL in a 100 mL capacity flask for each strain.
21. The flasks are agitated in the incubator shaker at 180 rpm and 25 °C for 15 min.
22. The contents of the flasks are distributed into three separate flasks for each strain, containing each one 5 mL.
23. In the first flask, no drug is added, as it is the negative untreated control. In the second, it was added concentration 1 (C1) and in the third, concentration 2 (C2) of the selected toxic. This process is repeated with the wild type strain and all the selected mutants.
24. All the flasks are shaked 15 min in the incubator shaker at 180 rpm at 25 °C.
25. The 96-well plate is filled with the contents of each flask, adding 200 µL/well starting from the wild type (WT) in the column 2, followed by WT+C1 in the column 3, WT+C2 in the column 4, and continuing with the mutants in the same order, respectively. Column 1 is filled with YES, functioning as the blank/solvent control. The remaining wells along the edges can be filled with YES or distilled water to avoid interferences. The experiments are conducted in triplicate.
26. The 96-well plate is placed on a plate shaker and agitated constantly at 1200 rpm for 16 h at 25 °C in a humidity chamber.
27. After 16 h exposure period, the density of the cell population is measured spectrophotometrically^5^ (Fig. 2).

**Fig. 2:**
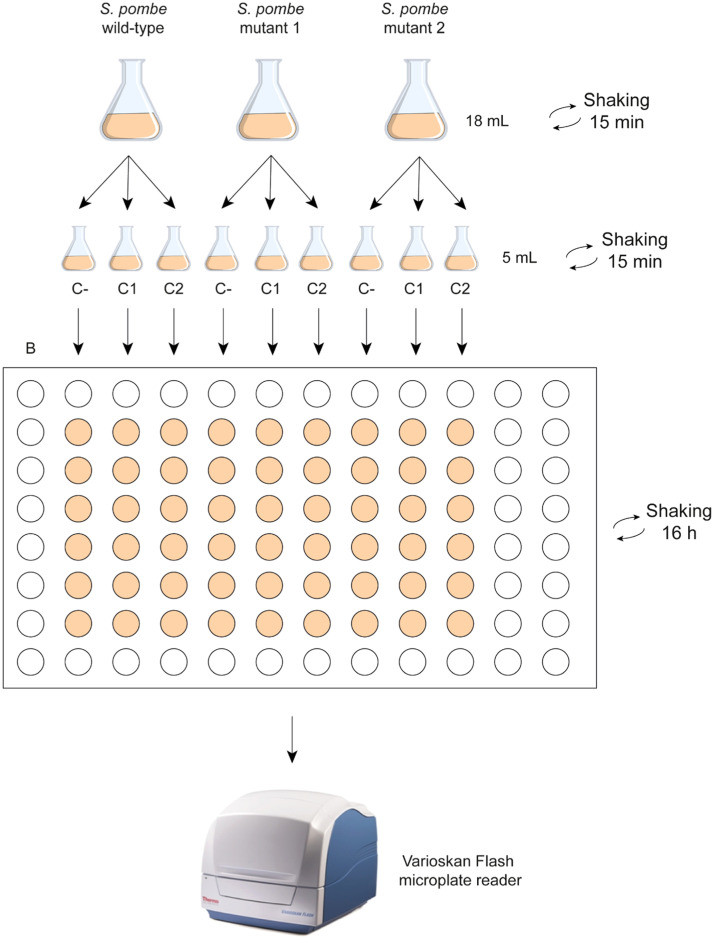
Investigation of the main mechanisms of action and detoxication by efflux pumps. Schematic illustrations of the steps 19–27 for the wild type strain and two mutants of *S. pombe*. B, C-, C1 and C2 indicate blank, negative control and the selected concentrations of the drug, respectively. If more mutants are selected to investigate the mechanisms of action or the efflux by pumps, other plates should be filled following the same scheme.

**Fig. 3:**
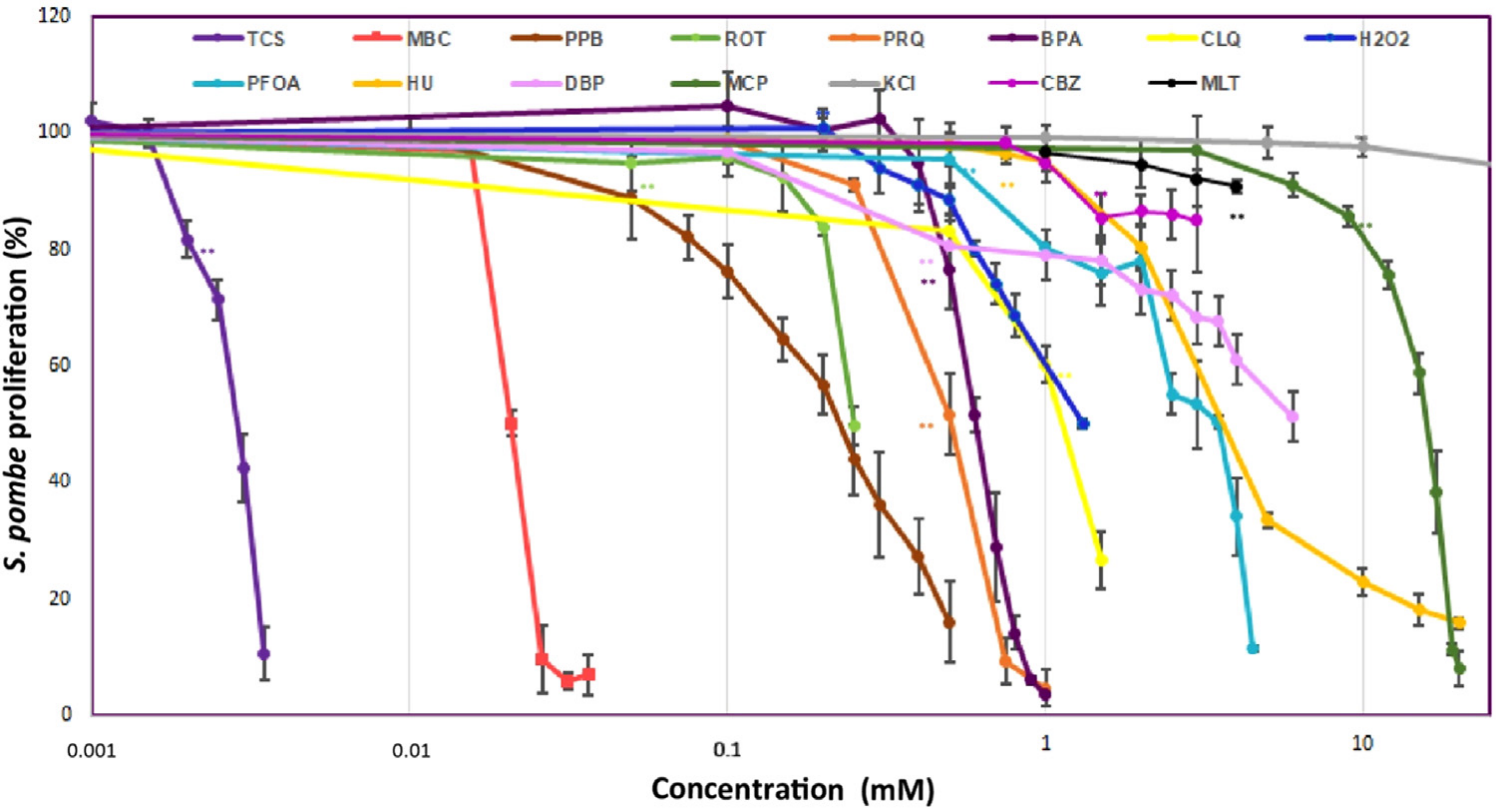
A: Inhibition of the proliferation of the wild type strain of *Schizosaccharomyces pombe* exposed for 20 h to triclosan (TCS)*, carbendazim (MBC), propylparaben (PPB)*, rotenone (ROT), paraquat (PRQ), chloroquine diphosphate (CLQ), H_2_O_2_, perfluorooctanoic acid (PFOA)*, hydroxyurea (HU), dibutyl phthalate (DBP)*, carbamazepine (CBZ), metoclopramide (MCP), melatonin (MLT) and KCl. Data expressed as% of unexposed controls (mean±SD). *Data taken from Alvarez Herrera et al., (2020). ** Indicates that from this value significant differences from control group were observed (*p* < 0.01).
28. The analysis of the results is conducted, considering that OD values correlate with the number of *S. pombe* cells. Experimental groups are statistically compared to the untreated cells of each tested concentration.
29. A dose-response regression is represented with Excel or other software [1,2].

## Method validation

For the dose-response assessment, the kind of graphic representation obtained follows the subsequent pattern in which the percentage of proliferation is represented on the x axis and concentration on the y axis in logarithmic scale, previous published in Álvarez-Herrera et al. [1].

In the case of the investigation of the main mechanism of action, triclosan has been selected as an example due to its high reproducibility. In the x axis shows the percentage of proliferation and the y axis, the concentration of triclosan selected, also previous published in Álvarez-Herrera et al. [2].

**Fig. 4:**
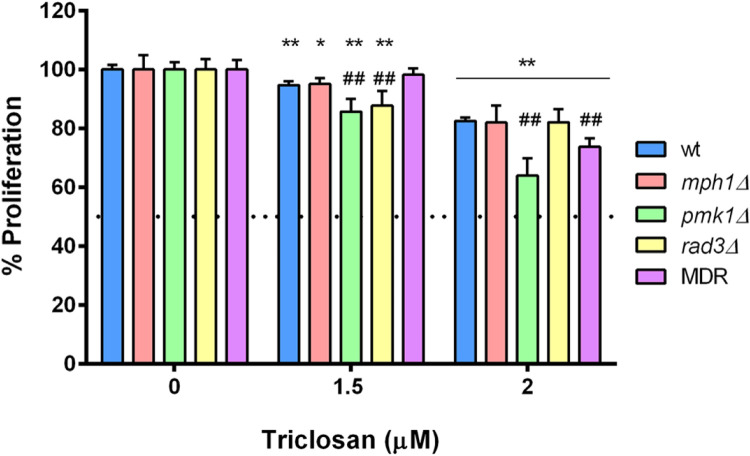
Investigation of the mechanisms of action studying the inhibition of the proliferation of the wild type*, mph1Δ, pmk1Δ, rad3Δ* and MDR-sup strains of *Schizosaccharomyces pombe*. A) exposed for 20 h in liquid media to 1.5 µM and 2 µM TCS. Significant differences between the treated defective strains and their unexposed controls are indicated by *p < 0.05 and **p < 0.01, and with the wild type treated groups are indicated by ##*p* < 0.01 (One-way ANOVA; Dunnett's test) [2].

The graphic representation in the investigation of detoxication by efflux pumps is similar to Fig. 4 and the only difference is the defective mutants used.

## Limitations

Two important aspects should to be taking into account to obtain reproducible data:

- It must be very precise in the exposure and measurement times, due to after the proposed time (16 h) the yeasts enter in the stationary phase.
- It is very important that the agitation on the plate-shaker was constantly at 1200 rpm.

## Ethics statements

No ethic statements to declare.

## Supplementary material *and/or* additional information [OPTIONAL]

None.

## CRediT authorship contribution statement

**Consuelo Álvarez-Herrera:** Investigation, Methodology, Data curation, Writing – original draft. **Sara Maisanaba:** Formal analysis, Visualization, Supervision, Writing – review & editing. **María Llana Ruíz-Cabello:** Investigation. **Guillermo Repetto:** Conceptualization, Funding acquisition, Supervision.

## Declaration of competing interest

The authors declare that they have no known competing financial interests or personal relationships that could have appeared to influence the work reported in this paper.

## Data Availability

Data will be made available on request.

## Footnotes

1 Note: In all cases, in order to assure adequate mixing and aeration, the volume of culture desired must be multiplied by 5 to determine the capacity of the flask used.

2 Note: It is crucial that the cultures attain the exponential growth phase, characterized by an optical density (OD) of 100 µL/well ranging from about 0.08 to 0.1 or a cell density of 5 × 10^5^ to 1 × 10^6^ cells/mL in the preparation and in the assay.

3 Note: The concentrations of drugs should be selected based on previous literature research.

4 Note: Avoid yeast deposits or bubbles that could affect the measurement.

5 Note: Ensure that there are no yeast deposits or bubbles that could affect the measurement.
